# Phenotypic discordance between primary and metastatic breast cancer in the large-scale real-life multicenter French ESME cohort

**DOI:** 10.1038/s41523-021-00252-6

**Published:** 2021-04-16

**Authors:** Thomas Grinda, Natacha Joyon, Amélie Lusque, Sarah Lefèvre, Laurent Arnould, Frédérique Penault-Llorca, Gaëtan Macgrogan, Isabelle Treilleux, Anne Vincent-Salomon, Juliette Haudebourg, Aurélie Maran-Gonzalez, Emmanuelle Charafe-Jauffret, Coralie Courtinard, Camille Franchet, Véronique Verriele, Etienne Brain, Patrick Tas, Cécile Blanc-Fournier, Agnès Leroux, Delphine Loussouarn, Anca Berghian, Eva Brabencova, Jean Pierre Ghnassia, Jean-Yves Scoazec, Suzette Delaloge, Thomas Filleron, Magali Lacroix-Triki

**Affiliations:** 1 Gustave Roussy Cancer Campus, Villejuif, France; 2 Institut Claudius Regaud, IUCT-Oncopôle, Toulouse, France; 3 Centre GF Leclerc, Dijon, France; 4 Centre Jean-Perrin, Clermont-Ferrand, France; 5 Institut Bergonié, Bordeaux, France; 6 Centre Léon Bérard, Lyon, France; 7 Institut Curie, Paris, France; 8 Centre Lacassagne, Nice, France; 9 ICM, Montpellier, France; 10 Institut Paoli-Calmettes, Marseille, France; 11 Unicancer R&D, Paris, France; 12 Institut de Cancérologie de l’Ouest, Angers, France; 13 Institut Curie, Saint-Cloud, France; 14 Atalante Pathologie, Rennes, France; 15 Centre François Baclesse, Caen, France; 16 Institut de cancérologie de Lorraine, Nancy, France; 17 Centre hospitalo-universitaire Nantes Hotel-Dieu, Nantes, France; 18 Centre Henri Becquerel, Rouen, France; 19 Institut Godinot, Reims, France; 20 Centre Paul Strauss, Strasbourg, France

**Keywords:** Breast cancer, Predictive markers

## Abstract

Expression of hormone receptor (HR) for estrogens (ER) and progesterone (PR) and HER2 remains the cornerstone to define the therapeutic strategy for breast cancer patients. We aimed to compare phenotypic profiles between matched primary and metastatic breast cancer (MBC) in the ESME database, a National real-life multicenter cohort of MBC patients. Patients with results available on both primary tumour and metastatic disease within 6 months of MBC diagnosis and before any tumour progression were eligible for the main analysis. Among the 16,703 patients included in the database, 1677 (10.0%) had available biopsy results at MBC diagnosis and on matched primary tumour. The change rate of either HR or HER2 was 27.0%. Global HR status changed (from positive = either ER or PR positive, to negative = both negative; and reverse) in 14.2% of the cases (expression loss in 72.5% and gain in 27.5%). HER2 status changed in 7.8% (amplification loss in 45.2%). The discordance rate appeared similar across different biopsy sites. Metastasis to bone, HER2+ and RH+/HER2- subtypes and previous adjuvant endocrine therapy, but not relapse interval were associated with an HR discordance in multivariable analysis. Loss of HR status was significantly associated with a risk of death (HR adjusted = 1.51, *p* = 0.002) while gain of HR and HER2 discordance was not. In conclusion, discordance of HR and HER2 expression between primary and metastatic breast cancer cannot be neglected. In addition, HR loss is associated with worse survival. Sampling metastatic sites is essential for treatment adjustment.

## Introduction

Breast cancer (BC) is the most prevalent malignancy, and metastatic breast cancer (MBC) the leading cause of cancer mortality among women in Western countries^1^. Around 5% of women diagnosed with breast cancer have synchronous metastases, while ~20% of those with early breast cancers will relapse and develop an incurable metastatic disease^2^. In both early and metastatic stages, therapeutic strategy and prognosis are highly dependent on the immunohistochemical evaluation of three major markers, estrogen receptor (ER), progesterone receptor (PR) and human epidermal growth factor receptor 2 (HER2). These markers are both the basis of the major breast cancer subtypes identification (with prognostic implications) and the targets of the main treatment strategies currently available^3^. In the past 20 years, several reports have highlighted the occurrence of ER, PR and HER2 expression changes between primary tumour and metastatic sites. The frequency of such HR/HER2 status modifications varies widely in the literature with reported discordance rates ranging from 10 to 56% for ER, 25 to 49% for PR and 3 to 16% for HER2^4–10^. Based on these results, most guidelines currently recommend re-biopsy of metastatic disease^11,12^. Recent and expanding knowledge regarding intra-tumour heterogeneity and time-dependent clonal selection during tumour progression and under therapeutic pressure has also called into question the necessity of re-biopsy metastatic sites to adapt treatment^13–16^. Biopsy of metastatic sites is a growingly available, globally safe technique, although it might be at risk of a few complications, including bleeding, infection, perforation, and unintended organ injury^17^. ESME (Epidemio-Strategy-Medical-Economical)-MBC is the largest available multicentre, nationwide, real-life, retrospective but prospectively maintained database of metastatic breast cancer patients, with a long follow-up^18,19^. The present study aimed at (i) comparing tumour immunophenotypic profiles between matched breast cancer primaries and metastatic sites in ESME-MBC, and (ii) assessing the impact of potential discordances on patient outcome.

## Results

### Study populations

**Fig. 1:**
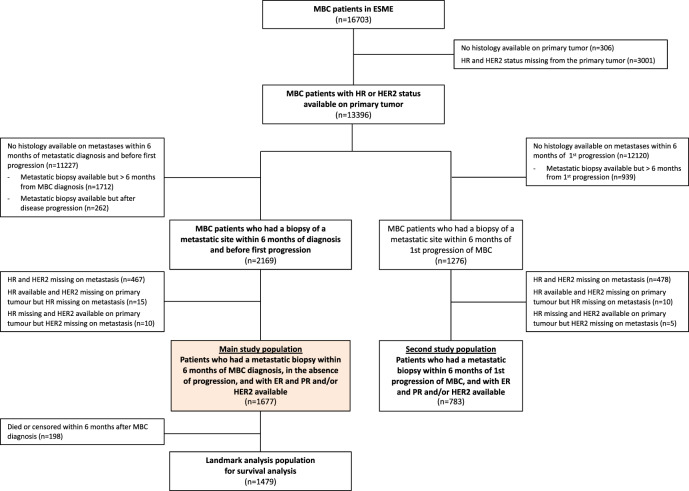
Flow chart.

From 2008/01/01 to 2014/12/31, 16,703 patients have been included in the ESME cohort. Histological reports of metastatic site biopsy were available for 8365 of them (50.1%), among whom 6391 (38.3%) and 5992 (35.9%) had HR and HER2 status available, respectively. Two thousands nine hundred thirty three patients (17.6%) had a metastatic biopsy performed at diagnosis or within the next 6 months, among whom 1677 (main study population, 10.0% of the whole cohort) had HR and/or HER2 status available on both MBC biopsy and primary tumour samples (see flow chart, Fig. 1).

At the time of first progression, 783 pts (second study population, 4.7% of the whole ESME cohort) had HR and/or HER2 status available on both MBC (within 6 months of the first progression) and primary tumour.

Table 1 describes the patient characteristics and the clinico-pathological features of the primary tumour and metastatic disease in the main study population, as compared to the global ESME population. With regards to phenotype, the majority of the population showed the HR+/HER2− phenotype (64.1% in the whole ESME population and 66.4% in the main study population), followed by triple negative phenotype (17.6% and 18.2%), HR+/HER2+ (10.7% and 9.4%) and finally the HR−/HER2+ phenotype (7.6% and 6%). In the study population, the prevalence of weak positive cases for ER (i.e. with 1–9% of stained cells) was low, accounting for 8/965 (0.8%) among cases with ER staining percentage values available on the primary tumor. Of these eight patients, only four had data available on metastases and one case (with 5% of ER+ cells on the primary tumor) showed a discordant ER status with 40% of ER+ cells on the metastasis. The prevalence of ER−/PR+ cases was also low (1.7% in the whole ESME population and 2.7% in the main study population, data not shown). Among patients in the main study population, 1324 (91%) received radiation therapy, 1042 (71.7%) received chemotherapy±-targeted therapy and 1029 (70.8%) endocrine therapy in the adjuvant setting.

**Table 1:** Clinical and histological features of the primary tumour and metastatic disease in the ESME cohort and main study population.

	Whole ESME population	Main study population		Whole ESME population	Main study population
	(*n* = 16,703)	(*n* = 1677)		(*n* = 16,703)	(*n* = 1677)
**At initial diagnosis**			Chemotherapy and/or targeted therapy		
Age: median (range)	54 (19: 98)	51 (23: 91)	No	3723 (30.7%)	411 (28.3%)
<50 years	6258 (37.6%)	730 (43.6%)	Yes	8410 (69.3%)	1042 (71.7%)
50–70 years	7883 (47.3%)	808 (48.2%)	Missing	18	2
>70 years	2517 (15.1%)	138 (8.2%)	Endocrine therapy		
Missing	45	1	No	4207 (34.7%)	425 (29.2%)
Sex			Yes	7930 (65.3%)	1029 (70.8%)
Male	149 (0.9%)	18 (1.1%)	Missing	14	1
Female	16554 (99.1%)	1659 (98.9%)	**At MBC diagnosis**		
Menopausal status			Age: median (range)	61 (19: 99)	60 (24: 93)
Yes	11,670 (69.9%)	1132 (67.5%)	Time to MBC		
No	4884 (29.2%)	527 (31.4%)	<6 months	4763 (28.6%)	226 (13.5%)
Not applicable	149 (0.9%)	18 (1.1%)	6–24 months	2186 (13.1%)	182 (10.9%)
Histological type			>24 months	9709 (58.3%)	1268 (75.7%)
Invasive carcinoma NST	11,902 (79.8%)	1306 (83.0%)	Missing	45	1
Invasive lobular	2088 (14.0%)	185 (11.8%)	Number of metastatic sites		
Other	917 (6.2%)	83 (5.3%)	1 site	9270 (55.5%)	667 (39.8%)
Missing	1796	103	2 sites	4017 (24.0%)	494 (29.5%)
ER status at diagnosis			≥3 sites	3416 (20.5%)	516 (30.8%)
Positive	9690 (75.0%)	1223 (75.5%)	Metastatic site of sampling		
Negative	3171 (24.5%)	384 (23.7%)		(*n* = 2933)	(*n* = 1677)
Heterogeneous	66 (0.5%)	13 (0.8%)	CNS/CSF	132 (4.6%)	42 (2.6%)
Missing	3776	57	Bone	692 (24.2%)	419 (25.5%)
PR status at diagnosis			Lung	258 (9.0%)	168 (10.2%)
Positive	7035 (55.8%)	908 (57.7%)	Lymph node	306 (10.7%)	169 (10.3%)
Negative	5435 (43.1%)	652 (41.4%)	Pleural	283 (9.9%)	121 (7.4%)
Heterogeneous	129 (1.0%)	13 (0.8%)	Skin	379 (13.3%)	203 (12.4%)
Missing	4104	104	Liver	514 (18.0%)	355 (21.6%)
HER2 status at diagnosis			Other	273 (9.5%)	151 (9.2%)
Positive	2070 (18.1%)	190 (14.7%)	Several	23 (0.8%)	14 (0.9%)
Negative	9187 (80.1%)	1064 (82.5%)	Missing	73	35
Equivocal	143 (1.2%)	26 (2.0%)	ER status on metastatic biopsy		
Heterogeneous	63 (0.5%)	9 (0.7%)	Positive	1601 (72.2%)	1163 (71.7%)
Missing	5240	388	Negative	617 (27.8%)	459 (28.3%)
Primary tumour subtype			Missing	715	55
TNBC	1897 (17.5%)	218 (18.1%)	PR status on metastatic biopsy		
HR+/ HER2−	6898 (64.1%)	795 (66.4%)	Positive	904 (42.1%)	669 (42.7%)
HR−/HER2+	821 (7.6%)	72 (6.0%)	Negative	1241 (57.9%)	899 (57.3%)
HR+/ HER2+	1148 (10.7%)	113 (9.4%)	Missing	788	109
Missing	5939	479	HER2 status on metastatic biopsy		
De novo MBC			Positive	342 (16.5%)	230 (15.3%)
Yes	4507 (27.1%)	221 (13.2%)	Negative	1670 (80.5%)	1230 (81.7%)
No	12151 (72.9%)	1455 (86.8%)	Equivocal	63 (3.0%)	45 (3.0%)
Missing	45	1	Missing	858	172
**Management of primary tumour (*n*** = **12,151)**			MBC subtypes		
			TNBC	356 (18.5%)	272 (19.3%)
Radiotherapy			HR+/HER2−	1251 (65.0%)	917 (65.2%)
No	1712 (14.1%)	131 (9.0%)	HR−/HER2+	150 (7.8%)	105 (7.5%)
Yes	10,426 (85.9%)	1324 (91.0%)	HR+/HER2+	168 (8.7%)	112 (8.0%)
Missing	13	0	Missing	1008	271

*NST* non-special type, *ER* Estrogen receptor expression, *PR* Progesterone receptor expression, *HER2* human epidermal growth factor receptor 2, *TNBC* Triple negative breast cancer, *CNS/CSF* Central nervous system, *CSF* cerebro-spinal fluid, *MBC* Metastatic breast cancer.

### HR and HER2 discordance between primary tumour and metastases, at metastatic diagnosis

**Fig. 2:**
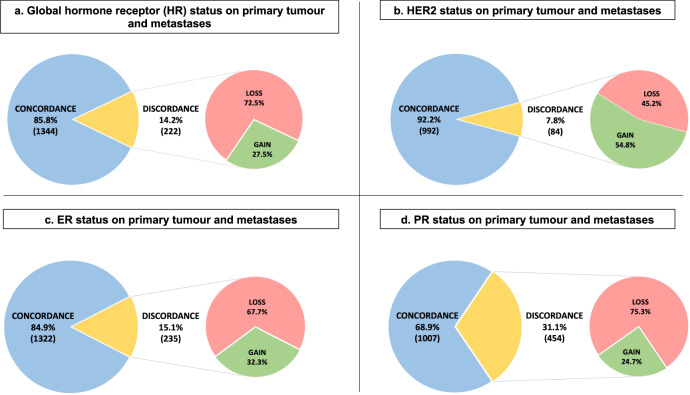
Modification of ER, PR and HER2 status between primary tumour and metastasis. **a** Hormone receptor status on primary tumour and metastasis (*n* = 1566), **b** HER2 status on primary tumour and metastasis (*n* = 1076), **c** Estrogen receptor status on primary tumour and metastasis (*n* = 1557), **d** Progesterone receptor status on primary tumour and metastasis (*n* = 1461). HR Hormone receptor expression, HER2 human epidermal growth factor receptor 2, ER Estrogen receptor expression, PR Progesterone receptor expression.

At metastatic diagnosis, the total discordance rate for HR and/or HER2 status between the primary tumour and metastases was 27.0% [95% CI 24.4–29.8]. The change rate for HR status was 14.2% [95% CI 12.5–16.0] with expression loss in 72.5% and expression gain in 27.5%. For ER status, 15.1% [95% CI 13.3–17.0] of cases showed a change with loss in 67.7% and gain in 32.3%. For PR status, a modification was observed in 31.1% [95% CI 28.7–33.5] with loss in 75.3% and gain in 24.7%. Finally, regarding the HER2 status, the modification rate was 7.8% [95% CI 6.3–9.6] with absence of overexpression/amplification in 45.2% and gain in 54.8% (Fig. 2).

**Fig. 3:**
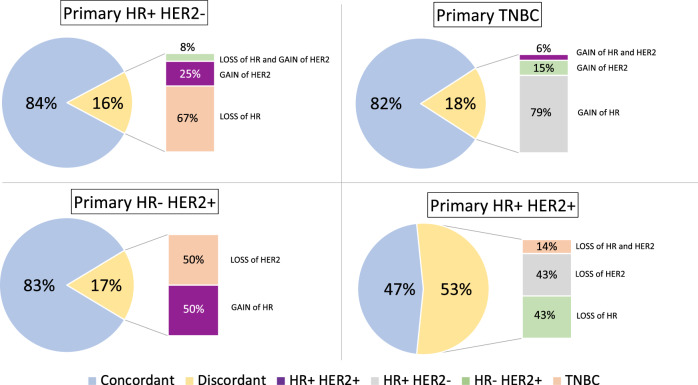
Breast cancer subtypes on primary tumour and metastatic disease. Primary HR+ HER2− (*n* = 641), Primary TNBC (*n* = 181), Primary HR−/HER2+ (*n* = 58), Primary HR+/HER2+ (*n* = 92). HR Hormone receptor expression, HER2 human epidermal growth factor receptor 2 status, TNBC Triple negative breast cancer.

**Fig. 4:**
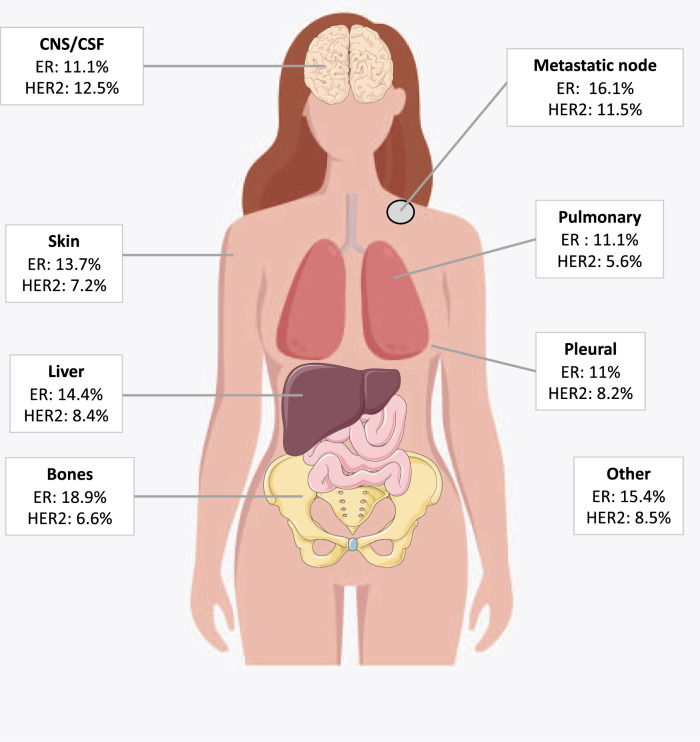
Phenotypic discordance by metastatic site. CNS central nervous system, CSF cerebro-spinal fluid, ER Estrogen receptor expression change, HER2 human epidermal growth factor receptor 2 expression change.

Among phenotipic subtypes, primary HR+/HER2+ tumours showed the highest rate of changes (53%), with 43% of HR loss, 43% of HER2 loss and 14% of both HR and HER2 loss (Fig. 3). Primary TNBC displayed a phenotypic change in 18% with a majority of HR gain (79%). The modification rates for HR−/HER2+ and HR+/HER2− subgroups were slightly lower (Fig. 3). The HR status change rate was globally similar across metastatic sites (from 10.1% in pleura to 16.9% in bone). HER2 change rate was 12.5% in central nervous system and 6.6% in bone sites (Fig. 4).

In the multivariable analysis, factors associated with HR discordance were metastasis to bone only (OR = 2.54, [95% CI 1.15–5.63], *p* = 0,022) compared to brain metastases, MBC subtypes HR+/HER2− (OR = 0.05, [95% CI 0.03–0.08], *p* < 0.001) and HER2+ (OR = 0.37, [95% CI 0.23–0.59], *p* < 0.001) compared to HR−/HER2− and primary tumour treatments with endocrine therapy (OR = 3.08, [95% CI 1.96–4.82], p < 0.001) (Table 2). Factors associated with HER2 discordance were MBC subtypes HR+/HER2− (OR = 0.45, [95% CI 0.21–0.98], *p* = 0.044) and HER2+ (OR = 5.73, [95% CI 2.83–11.60], *p* < 0.001) compared to HR−/HER2− and primary tumour treatments with endocrine therapy (OR = 2.95, [95% CI 1.47–5.91], *p* = 0.002). The year of diagnosis did not significantly impact HER2 discordance rate, albeit a trend for a decrease of discordance was observed after 2010 (HER2 discordance rate of 30/312 (9.6%) in 2007–2010, 41/595 (6.9%) in 2011–2013 and 13/169 (7.7%) in 2014, *p* = 0.3474).

**Table 2:** Multivariable analysis of factors associated with HR discordance.

	Odds ratio	(95% CI)	*p*-value
Age at first diagnosis			
<50 years	1.00		
≥50 years	1.11	(0.77: 1.59)	0.585
Time to MBC			
<6 months	1.00		
6–24 months	0.84	(0.35: 2.03)	0.703
>24 months	1.10	(0.50: 2.40)	0.814
Metastatic site			
Brain	1.00		
Visceral	1.78	(0.92: 3.44)	0.087
Non-visceral	1.38	(0.63: 3.01)	0.421
Bone only	2.54	(1.15: 5.63)	0.022
Number of metastatic site			
<3	1.00		
≥3	1.03	(0.68: 1.56)	0.884
MBC subtypes			
TNBC	1.00		
HR+/HER2−	0.05	(0.03: 0.08)	<0.001
HER2+	0.37	(0.23: 0.59)	<0.001
**Primary tumour treatments received**			
Chemotherapy or target therapy			
No	1.00		
Yes	0.98	(0.63: 1.53)	0.944
Hormonotherapy			
No	1.00		
Yes	3.08	(1.96: 4.82)	< 0.001
Year of diagnosis metastatic			
2007–2010	1		
2011–2013	0.90	(0.61: 1.33)	0.605
2014	0.80	(0.46: 1.41)	0.447

*MBC* Metastatic breast cancer, *HR* Hormone receptor expression, *HER2* human epidermal growth factor receptor 2.

### Impact of HR/HER2 status change on overall survival

**Fig. 5:**
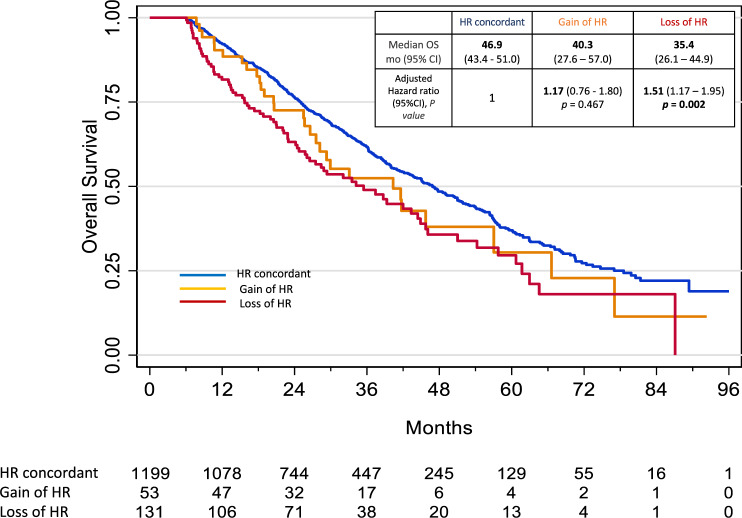
Overall survival according to HR modification status.

Of the 1677 patients analysed, 1479 were included in the survival analysis with a landmark approach at 6 months (198 patients with less than 6-month follow-up were excluded). After a median follow-up of 42.3 months [95%CI 40.1–44.8], the median of OS in was 45.1 months [95% CI 41.6–48.3]. After adjustment for age, histological grade, number and type of metastatic site in the multivariable analysis, HR discordance with loss of HR status was significantly associated with a worse OS (adjusted Hazard ratio = 1.51 [95% CI 1.17, 1.95] *p*-value = 0.002) and HR discordance with gain of HR was not significantly associated with OS (adjusted Hazard ratio = 1.17 [95%CI 0.76, 1.80] *p*-value = 0.467), compared to HR concordance (Supplementary Table 1, Fig. 5). In contrast, HER2 status discordance was not significantly associated with OS (Hazard ratio = 0.99 [95% CI 0.71; 1.38] and *p*-value = 0. 958).

### HR and HER2 discordance after the first progression

In second study population including patients who underwent a biopsy after the first progression (*n* = 783), the change rate of HR status between primary tumour and metastasis was 19.9% [95% CI 17–23] with expression loss in 79.9% and expression gain in 20.1%. For HER2 status, the modification rate was 10% [95% CI 7.6–12.9] with a loss of HER2 overexpression in 50.9% and a gain in 49.1%.

Again, the HR+/HER2+ subgroup showed the highest discordance rate: 58.2% of status modification, followed by TNBC (30.7%), HR−/HER2+ (28.2%) and HR+/HER2− (22.7%) (Supplementary Table 2).

## Discussion

The ESME-CSM platform is one of the largest real-life database for MBC, providing description of therapeutics and various ways of MBC management in France. ESME allowed the present very large evaluation of HR and HER2 discordance between primary tumour and metastatic disease. This study first establishes that, at MBC diagnosis, HR status changed (from positive = either ER or PR positive, to negative = both negative; and reverse) in 14.2% of the cases (expression loss in 72.5% and gain in 27.5%) and HER2 status in 7.8% (amplification loss in 45.2%). Factors associated with HR discordance are metastasis to bone, both HR+/HER2− and HER2 + MBC subtypes as well as endocrine therapy in adjuvant setting. For HER2, factors associated with discordance are both HR+/HER2− and HER2 + MBC subtypes as well as endocrine therapy in adjuvant setting. Finally, a discordance in HR with a loss of HR status leads to a reduction in overall survival in our study.

A recent meta-analysis reported discordance rates for ER, PR and HER2, of 19.3%, 30.9% and 10.3%, respectively^6^. In our study, the results were slightly lower, may be due to the discordance rate assessment at the first 6 months of metastatic diagnosis. Moreover, our discordance rates after the first progression were higher and closer to those reported in the literature, supporting the fact that phenotypic profile evolution may still occur during the course of metastatic progression. Another explanation could be that, in our study, the threshold for ER and PR positivity was ≥10% expression on tumour cells by immunohistochemistry. A threshold of ≥1%, as recommended by the ASCO/CAP guidelines^20^, may result in greater variability. However, the prevalence of cases with weak ER expression (i.e. in between 1 and 9%) is exceedingly low in the ESME cohort, accounting for only 0.8% of all histologies, in accordance with data from the literature^20^ and from French GEFPICS registry (1.4% among 14,000 invasive breast cancer, own unpublished data). Due to the small number of patients for whom the ER percentage values were available on the primary tumour and metastases, we could not perform a sensitivity analysis using a threshold of ≥1%. The difference in positivity threshold is therefore not likely to impact much the discordance rate. In addition, in real life, it might be hypothesized that patients with unexpected disease progression may have undergone more frequent biopsies, which could led to selection bias and might increase the discordance rate. In our study, the population does not differ from the whole ESME population, so this bias can be refuted.

Then, it is possible that some discordance in HER2 are explained by the so-called “equivocal status” in in situ hybridization which has disappeared in the new 2018 recommendations^21^. The change in the ASCO/CAP guidelines for HER2, which was published in 2013 and updated in the French GEFPICS guidelines in 2014^22^, is not likely to impact the HER2 discordance rate in our cohort, as the vast majority of the cases were sampled before 2013. Moreover, we did not observe any significant impact of time of diagnosis on HER2 discordance, albeit a trend to a decrease of discordance after 2010 was observed, probably reflecting some degree of improvement in HER2 IHC quality.

Although the exact mechanisms underlying phenotypic changes in MBC remain unknown, several explanations can be proposed. First, those discordances could be explained by a bias introduced by the performance of immunohistochemical assays used (sensitivity and specificity) and different sampling methods, like needle aspiration versus core biopsy, or surgical resection^23,24^. In addition, the decalcification step of bone samples makes immunohistochemistry less reliable and increases the risk of false negative results^25^. This may explain why bone metastases were a predictive factor of discordance in our study. Another explanation may be the bias linked to the different scoring methods used by pathologists. But even when an identical scoring method is used, reproducibility between pathologists may be suboptimal. Indeed, a significant discordance was observed between the primary tumour and recurrence under routine versus study conditions^26^. Nevertheless, generalization of guidelines and development of quality controls have greatly improved the reproducibility of IHC assays and their level of performance. Therefore, these technical limitations are not sufficient to explain the discordance rates observed in HR and HER2 status. The second and better explanation relies on intratumor and intertumor heterogeneity and the ability of tumours to generate tumour clones and subclones with different molecular properties, either spontaneously or following the selection pressure imposed by the treatments^13^.

The ESME-CSM platform provides a large database representing real-life practice at the scale of a country, allowing to answer or generate some research questions. Such large cohorts are useful to provide data on uncommon subtypes or phenotypes. For example, the prevalence of ER−/PR+ cases in this database is low (*n* = 213, 1.7% in the whole ESME cohort), as reported in the literature with a prevalence ranging from 0.3 to 3.4%^27–29^.The ER−/PR+ phenotype is still a controversial molecular subtype, as some data suggest that this phenotype might be mainly due to technical artifacts^20,30^, while other studies confirmed it as a true but rare biologic subtype^27–29^. Moreover, the largest studies to date on this rare phenotype reported a trend for a poorer prognosis (early recurrence, poorer overall and disease free survival) as compared with ER+/PR+ tumors, and more similar to ER-/PR- tumors^28,29^. Albeit we cannot rule out that some of these cases in the ESME cohort might be due to a technical artifact, further exploration of these 213 cases might be of interest.

Nevertheless, the analysis of the ESME-CSM platform reveals that a certain number of data are missing. With regard to metastatic disease, few samples are available, with only 17.6% patients having a referenced histological result at MBC diagnosis. This lack of information can be explained by the fact that during data collection (2008–2013), the international guidelines did not yet recommend performing a biopsy of the metastatic site^31^. Additionally, metastatic biopsy cannot be reasonably performed systematically, for multiple reasons (contraindication to biopsy procedure, patient’s refusal or inaccessible site). In addition, heterogeneous tumours at first diagnosis were considered as missing data. Although such cases represent less than 3%, our population was therefore not fully representative of breast cancer patients. But from a practical point of view, our results support the necessity to perform biopsies of metastatic sites for the management of MBC for several reasons: to definitively establish the diagnosis of metastatic disease, to assess the prognostic value of metastatic subtype, to guide the choice of an appropriate therapy (so as not to miss an indication of targeted therapy) and finally, to assess of emerging biomarkers, which grant access to new treatments^32^. This practice is particular necessary in the RH+/HER2+ subgroup, which appears to be the most unstable.

Finally, we observed a statistically significant association between HR discordance (especially for HR loss) and survival, whilst this was not the case for HER2 conversion. This observation could be due to the lower incidence of discordant HER2 status (only 7.8% of the cases, *n* = 84), leading to insufficient statistical power. However, our results on this aspect are similar to those reported in a recent publication^33^. In this single center cohort including 390 invasive breast cancers, the authors reported an overall discordance rate of 18.3% for ER, 40.3% for PR and 13.7% for HER2. Despite the higher incidence of HER2 discordance in their study, the authors failed to demonstrate any association between HER2 conversion and survival, while such an association was observed for ER conversion in a way akin to our data. Other factors must therefore exist to explain the lack of impact of a discordant HER2 status on survival. One could hypothesize that the conversion of HER2 status might correspond to a late oncogenic event, in cases with *HER2* gene status near the positivity threshold, as opposed to the early, driver oncogenic event of *HER2* amplification observed in HR−/HER2+ subtype. Indeed, HER2 equivocal cases are often highly heterogeneous in terms of genetic subclones, and the vast majority are HR+. The fact that HR+/HER2+ subgroup is the subtype showing the highest rate of changes in our study would support this hypothesis. In other terms, the lack of impact of a conversion of HER2 status on survival might reflect a passenger event quite different from the *HER2* oncogenic addiction.

In conclusion, in this large-scale real-life setting, a change of HR and HER2 expression between primary BC and matched MBC was observed in 14.2% and 7.8% of cases, respectively, in the first 6 months of metastatic diagnosis. With regards to molecular subtype, 53% of the primary HR+/HER2+ tumour changed their status. In addition, a loss of HR is associated with worse survival and discordance rates were higher after the first progression. In conclusion, the evaluation of HR and HER2 status remains essential for MBC treatment tailoring.

## Methods

### ESME database

The ESME-MBC (NCT03275311) cohort is an ongoing national cohort collecting real-life information from all consecutive MBC patients aged ≥18 year-old who initiated their MBC treatment in one of the 18 French Comprehensive Cancer Centers^19^. Data collected include patient and tumour characteristics at primary and metastatic settings, outcomes and treatment patterns. All data are updated annually. For the present study, we used data collected for MBC patients who entered the cohort from 2008/01/01 to 2014/12/31.

### Objectives

The primary objective of this study was to describe the discordance of hormone receptors (HR) and HER2 status between primary tumours and matched metastases, on samples collected within 6 months from MBC diagnosis, and before any progression.

Secondary objectives were to search for factors predicting for HR and HER2 discordances, to evaluate whether HR and HER2 discordance had a prognostic impact on overall survival, and finally, to evaluate the evolution of HR and HER2 discordance over time, after the first progression.

### Definitions

All immunohistochemistry (IHC) assessments were performed locally as per routine practice in each institution and were reported in the central database. All 18 Comprehensive Cancer Centres used the same guidelines (i.e. updated ASCO-CAP recommendations and their adaptation issued by the French GEFPICS Group) for tumour testing regarding ER, PR and HER2^22,34^, and were participating to mandatory external proficiency tests performed each year in the frame of quality assurance programs (AFAQAP, UKNEQAS). Tumours were reported as ER-positive and PR positive, respectively, if ER and PR expression was observed in ≥10% of tumour cells by IHC, following French guidelines^35^. A global HR-positive (HR+) status was considered if ER and/or PR were expressed. A global HR-negative (HR−) status was defined by absence of both ER and PR detectable expressions. HER2-positive (HER2+) breast cancer was defined by a 3+ HER2 IHC score, or a 2+ IHC score associated with *HER2* gene amplification by in situ hybridization. Multifocal heterogeneous tumors showing a different status for a given biomarker (either HR or HER2) between the different primary tumors were excluded from the analysis only for this biomarker. The primary tumour status was defined on the first surgical histology sample available. In the absence of surgery of the primary tumour, pathological data from the initial core needle biopsy were selected. The first metastatic HR/HER2 status was obtained on the first available sample within the first 6 months of metastatic diagnosis and prior to any tumour progression. When available, the second metastatic HR/HER2 status was obtained on histology sample within the first 6 months after first progression. Global HR status was considered as discordant if the primary tumour was positive (ER and/or PR positive) and the metastasis negative (ER and PR negative); or reverse. The same rules were applied to HER2 status.

### Definition of subtypes

Three breast cancer subtypes were defined based on ER, PR and HER2 status, as used in other ESME publications (18): HR+/HER2− subtype was defined by hormone receptor-positive (either ER or PR positive) and HER2− status, HER2+subtype by HER2 positivity as assessed by IHC and in situ hybridization in case of 2+ IHC score, and triple negative (TNBC) subtype by lack of expression of ER, PR and HER2.

### Study population

For the primary objective and the first secondary objectives of the present study, patients were eligible if they had at least one histological report with HR or HER2 status on primary tumour and at least one histological report with HR or HER2 status on a metastasis within the first 6 months of MBC diagnosis, before any disease progression (main study population). For the other secondary objective, patients were included if they had histological reports and HR or HER2 status on primary tumour and metastasis within 6 months from the first progression of MBC (second study population).

### Ethics approval

The present analysis was approved by an independent ethics committee (Comité De Protection Des Personnes Sud-Est II- 2015-79). No formal dedicated informed consent was required but all patients had approved the re-use of their electronically recorded data. In compliance with French regulations, the ESME-MBC database was authorized by the French data protection authority (Registration ID 1704113 and authorization N°DE-2013.−117). Moreover, in compliance with the applicable European regulations, a complementary authorization was obtained on 2019 regarding the ESME research Data Warehouse.

### Statistical analyses

Data were described using frequencies and percentages for qualitative variables and using median and range for quantitative variables. The number of missing data was presented for each variable, but not considered for percentage calculations. The 95% confidence intervals of discordance rate were calculated using exact binomial distribution.

Univariable analyses of factors potentially associated with HR and HER2 discordance were performed using the Chi-squared test or the Fisher exact test for qualitative. Multivariable analyses of phenotypic discordance were performed using logistic regression models. The Odds Ratio (OR) and 95% confidence interval (95%CI) were presented for each variable. Variables of interest were age at diagnosis of MBC (< or ≥50 years); time to MBC defined as the time from the diagnosis of the primary cancer to the one of MBC (<6, [6–24], >24 months); metastatic sites (bone only, bone and non-visceral metastases [skin, lymph nodes…], visceral metastases, brain metastases); number of metastatic sites (<3, ≥3); MBC subtypes (HR+/HER2−, HER2+ and HR−/HER2−), primary tumour treatments received.

Overall survival (OS) was estimated using the Kaplan-Meier method and measured as the time from diagnosis of metastatic disease to death due to any cause or last contact (censored data). Comparisons between groups were estimated using the log-rank test and a multivariable analysis was performed using Cox proportional hazard model. The Hazard Ratio and 95% confidence interval (95%CI) were presented for each variable. All variables significant in univariable analysis were included in multivariable analysis. Survival analysis was performed using a landmark approach to avoid the guarantee-time bias. Thus, patients who died or were censored before the landmark were excluded. The landmark was chosen at 6 months, date on which the first metastatic HR/HER2 status was defined.

All statistical tests were two-sided and a *p*-value <0.05 was considered statistically significant. All analyses were performed using Stata version 13 (StataCorp LP, College Station, TX).

### Reporting summary

Further information on research design is available in the Nature Research Reporting Summary linked to this article.

## Supplementary information

Supplement Table 1 & 2

Reporting Summary

## Data Availability

The data generated and analysed during this study are described in the following data record: 10.6084/m9.figshare.14248691^36^. All data are contained in the ESME database, which is managed by Unicancer (http://www.unicancer.fr/). However, the ESME database is not publicly available for the following reason: in the ESME Research program, public data sharing is not automatic in order to ensure that only trained users can analyse the ESME datasets. The analysis datasets will be made available only under data transfer and use agreements executed between Unicancer, ICR (https://www.icr.ac.ukx/) and the potential licensee. Interested parties should contact the corresponding author.
