# Supplementary material for: Phenotypic discordance between primary and metastatic breast cancer in the large-scale real-life multicenter French ESME cohort
Source: NPJ Breast Cancer. 2021 Apr 16;7:41. doi: 10.1038/s41523-021-00252-6 (PMC8052407; doi:10.1038/s41523-021-00252-6)
Supplement: Supplementary file 1 — Supplement Table 1 & 2 [file 41523_2021_252_MOESM1_ESM.pdf]

Phenotypic discordance between primary and metastatic breast cancer in the large scale real-life multicenter French ESME cohort, Grinda et al.
Online-only supplements

Supplement Table 1: Multivariable analysis for overall survival including HR discordance

Multivariable analysis for overall survival including HR discordance			
	Hazard Ratio	(95% CI)	p-value
Hormone receptor discordance			
Concordant	1.00		
Discordant with gain of HR	1.17	(0.76 : 1.80)	0.467
Discordant with loss of HR	1.51	(1.17 : 1.95)	0.002
Age at first diagnosis			
< 50 years	1.00		
≥ 50 years	1.21	(1.03 : 1.43)	0.022
Histological grade			
Grade I/II	1.00		
Grade III	1.96	(1.66 : 2.31)	< 0.001
Number of metastatic site			
< 3	1.00		
≥ 3	1.33	(1.10 : 1.62)	0.004
Metastatic site			
Visceral	1.00		
Non visceral	0.73	(0.60: 0.88)	0.001
HR: Hormone receptor expression			

Supplement Table 2: Comparison of HR and HER2 status between primary tumour and metastatic disease when biopsy occurs after 1st progression

	Status of metastastic disease after first progression			
	N= 473			
	TNBC	HR+/HER2-	HR-/HER2+	HR+/HER2+
Status of PT				
TNBC	52 (69.3%)	18 (24.0%)	5 (6.7%)	0 (0%)
HR+/HER2-	51 (16.8%)	235 (77.3%)	4 (1.3%)	14 (4.6%)
HR-/HER2+	3 (7.7%)	3 (7.7%)	28 (71.8%)	5 (12.8%)
HR+/HER2+	6 (10.9%)	13 (23.6%)	13 (23.6%)	23 (41.8%)
HR: Hormone receptor expression, HER2: human epidermal growth factor receptor 2, TNBC: Triple negative breast cancer				
