# Supplementary material for: Phenotypic discordance between primary and metastatic breast cancer in the large-scale real-life multicenter French ESME cohort
Source: NPJ Breast Cancer. 2021 Apr 16;7:41. doi: 10.1038/s41523-021-00252-6 (PMC8052407; doi:10.1038/s41523-021-00252-6)
Supplement: Supplementary file 2 — Reporting Summary [file 41523_2021_252_MOESM2_ESM.pdf]

nature research

Corresponding author(s): Grinda Thomas

Last updated by author(s): Feb 16, 2021

Reporting Summary

Nature Research wishes to improve the reproducibility of the work that we publish. This form provides structure for consistency and transparency in reporting. For further information on Nature Research policies, see our Editorial Policies and the Editorial Policy Checklist.

Statistics

For all statistical analyses, confirm that the following items are present in the figure legend, table legend, main text, or Methods section.

n/a	Confirmed	
☐	☒	The exact sample size (*n*) for each experimental group/condition, given as a discrete number and unit of measurement
☐	☒	A statement on whether measurements were taken from distinct samples or whether the same sample was measured repeatedly
☐	☒	The statistical test(s) used AND whether they are one- or two-sided<br>*Only common tests should be described solely by name; describe more complex techniques in the Methods section.*
☐	☒	A description of all covariates tested
☐	☒	A description of any assumptions or corrections, such as tests of normality and adjustment for multiple comparisons
☐	☒	A full description of the statistical parameters including central tendency (e.g. means) or other basic estimates (e.g. regression coefficient) AND variation (e.g. standard deviation) or associated estimates of uncertainty (e.g. confidence intervals)
☐	☒	For null hypothesis testing, the test statistic (e.g. *F*, *t*, *r*) with confidence intervals, effect sizes, degrees of freedom and *P* value noted<br>*Give P values as exact values whenever suitable.*
☒	☐	For Bayesian analysis, information on the choice of priors and Markov chain Monte Carlo settings
☒	☐	For hierarchical and complex designs, identification of the appropriate level for tests and full reporting of outcomes
☒	☐	Estimates of effect sizes (e.g. Cohen's *d*, Pearson's *r*), indicating how they were calculated

Our web collection on statistics for biologists contains articles on many of the points above.

Software and code

Policy information about availability of computer code

Data collection	The ESME-MBC (NCT03275311) cohort is an ongoing national cohort collecting real-life information from all consecutive MBC patients aged > 18 year-old who initiated their MBC treatment in one of the 18 French Comprehensive Cancer Centers19. Data collected include patient and tumour characteristics at primary and metastatic settings, outcomes and treatment patterns. All data are updated annually. For the present study, we used data collected for MBC patients who entered the cohort from 2008/01/01 to 2014/12/31.
Data analysis	All analyses were performed using Stata version 13 (StataCorp LP, College Station, TX).

For manuscripts utilizing custom algorithms or software that are central to the research but not yet described in published literature, software must be made available to editors and reviewers. We strongly encourage code deposition in a community repository (e.g. GitHub). See the Nature Research guidelines for submitting code & software for further information.

Data

Policy information about availability of data

All manuscripts must include a data availability statement. This statement should provide the following information, where applicable:

- Accession codes, unique identifiers, or web links for publicly available datasets
- A list of figures that have associated raw data
- A description of any restrictions on data availability

Data availability :
Data supporting the findings of this study are available from Unicancer, but restrictions apply to the availability of this data, which was used under licence for this study, and is therefore not publicly available. However, the data are available from the authors upon reasonable request and with permission from Unicancer.

Field-specific reporting

Please select the one below that is the best fit for your research. If you are not sure, read the appropriate sections before making your selection.

☒ Life sciences ☐ Behavioural & social sciences ☐ Ecological, evolutionary & environmental sciences

For a reference copy of the document with all sections, see nature.com/documents/nr-reporting-summary-flat.pdf

Life sciences study design

All studies must disclose on these points even when the disclosure is negative.

Sample size	The ESME-MBC (NCT03275311) cohort is an ongoing national cohort collecting real-life information from all consecutive MBC patients aged > 18 year-old who initiated their MBC treatment in one of the 18 French Comprehensive Cancer Centers19. Data collected include patient and tumour characteristics at primary and metastatic settings, outcomes and treatment patterns. All data are updated annually. For the present study, we used data collected for MBC patients who entered the cohort from 2008/01/01 to 2014/12/31.
Data exclusions	For the primary objective and the first secondary objectives of the present study, patients were eligible if they had at least one histological report with HR or HER2 status on primary tumour and at least one histological report with HR or HER2 status on a metastasis within the first 6 months of MBC diagnosis, before any disease progression (main study population). For the other secondary objective, patients were included if they had histological reports and HR or HER2 status on primary tumour and metastasis within 6 months from the first progression of MBC (second study population).
Replication	*Describe the measures taken to verify the reproducibility of the experimental findings. If all attempts at replication were successful, confirm this OR if there are any findings that were not replicated or cannot be reproduced, note this and describe why.*
Randomization	*Describe how samples/organisms/participants were allocated into experimental groups. If allocation was not random, describe how covariates were controlled OR if this is not relevant to your study, explain why.*
Blinding	*Describe whether the investigators were blinded to group allocation during data collection and/or analysis. If blinding was not possible, describe why OR explain why blinding was not relevant to your study.*

Reporting for specific materials, systems and methods

We require information from authors about some types of materials, experimental systems and methods used in many studies. Here, indicate whether each material, system or method listed is relevant to your study. If you are not sure if a list item applies to your research, read the appropriate section before selecting a response.

Materials & experimental systems

n/a	Involved in the study	
☒	☐	Antibodies
☒	☐	Eukaryotic cell lines
☒	☐	Palaeontology and archaeology
☒	☐	Animals and other organisms
☐	☒	Human research participants
☐	☒	Clinical data
☒	☐	Dual use research of concern

Methods

n/a	Involved in the study	
☒	☐	ChIP-seq
☒	☐	Flow cytometry
☒	☐	MRI-based neuroimaging

Human research participants

Policy information about studies involving human research participants

Population characteristics	The ESME-MBC (NCT03275311) cohort is an ongoing national cohort collecting real-life information from all consecutive MBC patients aged > 18 year-old who initiated their MBC treatment in one of the 18 French Comprehensive Cancer Centers. Data collected include patient and tumour characteristics at primary and metastatic settings, outcomes and treatment patterns. All data are updated annually. For the present study, we used data collected for MBC patients who entered the cohort from 2008/01/01 to 2014/12/31.<br>For the primary objective and the first secondary objectives of the present study, patients were eligible if they had at least one histological report with HR or HER2 status on primary tumour and at least one histological report with HR or HER2 status on a metastasis within the first 6 months of MBC diagnosis, before any disease progression (main study population). For the other secondary objective, patients were included if they had histological reports and HR or HER2 status on primary tumour and metastasis within 6 months from the first progression of MBC (second study population).
Recruitment	see above

Ethics oversight	The present analysis was approved by an independent ethics committee (Comité De Protection Des Personnes Sud-Est II-2015-79). No formal dedicated informed consent was required but all patients had approved the re-use of their electronically recorded data. In compliance with French regulations, the ESME MBC database was authorized by the French data protection authority (Registration ID 1704113 and authorization N°DE-2013.-117). Moreover, in compliance with the applicable European regulations, a complementary authorization was obtained on 2019 regarding the ESME research Data Warehouse.

Note that full information on the approval of the study protocol must also be provided in the manuscript.

Clinical data

Policy information about clinical studies

All manuscripts should comply with the ICMJE guidelines for publication of clinical research and a completed CONSORT checklist must be included with all submissions.

Clinical trial registration	Clinical Trial: Yes<br>Clinical Trial Registry Name: Clinicaltrials.gov (http://www.clinicaltrials.gov)<br>Clinical Trial Registry URL: https://clinicaltrials.gov/ct2/show/NCT03275311<br>Clinical Trial Registry Number: NCT03275311
Study protocol	Retrospective study
Data collection	ESME data collection
Outcomes	The primary objective of this study was to describe the discordance of hormone receptors (HR) and HER2 status between primary tumours and matched metastases, on samples collected within 6 months from MBC diagnosis, and before any progression. Secondary objectives were to search for factors predicting for HR and HER2 discordances, to evaluate whether HR and HER2 discordance had a prognostic impact on overall survival, and finally, to evaluate the evolution of HR and HER2 discordance over time, after the first progression.
